# An artificial intelligence method for comprehensive evaluation of preschool education quality

**DOI:** 10.3389/fpsyg.2022.955870

**Published:** 2022-07-28

**Authors:** Peilin Niu

**Affiliations:** School of Education Science, Yulin Normal College, Yulin, China

**Keywords:** fuzzy neural network, early childhood, preschool education, comprehensive quality evaluation, quality of teaching

## Abstract

The evolution in the quality of teaching for preschool education is worth studying. In this article, we solved the qualitative problems in the comprehensive quality evaluation by suggesting a method of quantitative combination and establishing a set of indicators suitable for the comprehensive quality evaluation of students in the kindergarten. According to the experience summed up by previous scholars, the weight of each index is obtained by an analytic hierarchy process. This study analyzed the defects and causes of fuzzy comprehensive evaluation and the neural network model in the construction of early childhood and preschool education's comprehensive quality evaluation model and propose a Feedforward Neural Network (FNN) model. FNN combined with neural network (NN) and fuzzy logic characteristics introduces fuzzy concepts and fuzzy inference rules into neural networks of neurons, the connection power, and network learning. It improves the learning ability of NN and fuzzy evaluation of the power of expression and effectively exerts the advantages of fuzzy logic and neural network to make up for their shortcomings. However, the convergence speed is very slow. To solve this problem, the similarity measure was used to improve the number of hidden layer nodes of the network. The effectiveness and feasibility of the FNN improved hidden layer nodes are verified by an example so as to realize the automation of comprehensive quality evaluation.

## Introduction

The pressure of exam-oriented education continues to be passed down. This not only intensifies the general exam-oriented tendency of basic education but also causes a misunderstanding of “evaluation is only examination.” As a result, the healthy development and process of teachers' evaluation have not been paid due attention. It appears that the teacher's responsibility is to teach; however, teaching and evaluation are two completely separate functions. In the preschool education stage, because there is no external unified examination, the evaluation by preschool teachers is mostly ignored. To a large extent, this situation is rooted in the one-sided view of exam-oriented teaching and evaluation represented by “evaluation of learning.” In fact, besides judging the value of learning results, teaching evaluation also plays an important role in promoting learning, that is, “evaluation of promoting learning.” Only by unifying “evaluation of learning” and “evaluation of promoting learning” and forming a complete concept of teaching evaluation can we better understand and develop the evaluation quality of front-line teachers, especially kindergarten teachers. The evaluation quality of preschool teachers can be improved if the focus is placed on the process of promoting children's learning and growth, which is essentially different from the exam-oriented evaluation model. The paradigm shift from “evaluation of learning” to “evaluation of promoting learning” requires preschool teachers to focus on developing the ability to collect evaluation-relevant information based on observation, to analyze domain content knowledge and children's learning characteristics, and to evaluate based on domain teaching knowledge. Also, through the knowledge of evaluation, skills learning, professional research, practice and reflection, cooperative discussion, independent development, and similar paths, teachers can gradually enrich and enhance their evaluation quality, resulting in better integration of teaching and evaluation. This study focuses mainly on the following innovation points: (1) we addressed the qualitative problems in the comprehensive quality evaluation with the method of quantitative combination and established a set of indicators suitable for the comprehensive quality evaluation of students in the kindergarten of preschool education combination; (2) we analyzed the defects and causes of fuzzy comprehensive evaluation and neural network model in the construction of early childhood and preschool education's comprehensive quality evaluation model and propose an FNN model; and (3) we combined subjective and objective evaluation factors, indexed them based on the analytic hierarchy process obtained in this study, and suggested the automated model of comprehensive quality evaluation.

## Literature review

Data mining (DM) is a process of discovering hidden patterns and knowledge from massive data through certain algorithms (Han and Kamber, 2001), which has been mostly applied in so many fields. With the development of education informationization, the wisdom of campus construction and education data growing exponentially, EDM (education DM) arises at a historic moment. It is designed to analyze the education environment to solve the problems of education research and unique data (Baker, 2010; Baker and Siemens, 2014). EDM can be understood as the application of DM in education big data. It is not only the embodiment of digital education research, but it is also an inevitable demand for the development of education informationization (Li Ting and Gangshan, 2010). EDM is an interdisciplinary field combining computer science, statistics, and pedagogy (Romero and Ventura, 2013). Non-Chinese studies on EDM started earlier, with relevant papers published on the Web of Science as early as 1995 and a series of reviews published thereafter (Romero and Ventura, 2007, 2013; Baker and Yacef, 2009; US Department of Education, 2012; Peña-Ayala, 2014; Dutt et al., 2017; Bakhshinategh et al., 2018). A data mining survey (Romero and Ventura, 2007) by Romero and Ventura summarized the research results of EDM from 1995 to 2005. Another review of data mining by Baker and Yacef (2009) summarized the work of early EDM and foretold that EDM will have greater influence in the field of education in the future. In 2012, the US Department of Education released Promoting Teaching and Learning through Educational Data Mining and Learning Analysis, which introduced the application status and existing problems of EDM in the US education field in detail (US Department of Education, 2012) and attracted more researchers to devote themselves to the EDM field. In the study of Romero and Ventura (2013), the background, the mining process, the common methods, and the development process of EDM were comprehensively introduced. Peña-Ayala (2014) analyzed about 240 EDM application cases published between 2010 and 2013 and found that most EDM application studies were based on three parts, namely, educational tasks, methods, and algorithms. Dutt et al. (2017) undertook a systematic literature review of clustering algorithms and their applicability and availability in EDM for 30 years (1983–2016) and pointed out that the development of semi-supervised clustering algorithm was the next research direction. Bakhshinategh et al. (2018) reviewed the development of EDM from 2010 to 2017, divided its application scenarios into several categories, and introduced some representative examples for each category.

As early as 1864, the British G. Fisher formed the Set of Homework Scales as a reference standard for teachers to evaluate students' scores in various subjects. Later, Wilford M. Aikin, an American, led the “Eight-Year Study,” a famous middle school curriculum reform movement in the history of American education, from 1932 to 1940 (Saaty, 1980). In his book, “Reflecting on the Eight-Year Study,” Ralph W. Tyler put forward the principle of evaluation activities, described the concept of educational tests, introduced the principles and methods of educational evaluation activities by using educational tests and other methods, and formed the “behavior objective evaluation model” (Fresko and Nasser, 2001). Since then, various new evaluation models, for e.g., D. L. Stufflebeam's CIPP model and M. Scriven's target dissociation model, and theories have been put forward, developed, and improved continuously (Bellman and Zadeh, 1970; Tamura et al., 1971).

With the development of neural grid and machine learning models (Jain et al., 2021; Mohan et al., 2021; Gupta et al., 2021), traditional evaluation methods (principal component analysis (Ezazipour and Golbabai, 2020), fuzzy comprehensive evaluation (Wold et al., 1987), hierarchical analysis (Bing et al., 2010), and entropy weight method (Ho, 2008) and the introduction of the comprehensive evaluation system of the machine learning method have become a hot research topic.

In this study, we focused on the establishment of the overall quality of early childhood education contents, index, and index weight. Finally, the distribution of the comprehensive evaluation method is discussed to establish a model from a multidisciplinary perspective, which combined qualitative and quantitative analysis and met the requirements of the era and preschool education employment marketization, met the scientific quality of preschool education, and matched the system of the comprehensive quality evaluation system.

## The construction of the comprehensive quality evaluation index system for early childhood and preschool education and the determination of weightage

Today, with the comprehensive development of quality education, our understanding and research on early childhood and preschool education's comprehensive quality are at a stage of continuous development and perfection. Although most kindergarten schools have implemented the comprehensive quality evaluation of early childhood and preschool education, the main evaluation standard is still the students' examination results, which is not fully reflected in measuring the ideological and political quality, innovative spirit, and practical ability of early childhood and preschool education. Therefore, only by establishing a sound evaluation index system, collecting data in a scientific and reasonable way, and determining reasonable weightage can we ensure objective and fair evaluation results, guide early childhood and preschool education to cultivate a variety of qualities and abilities, and promote the development of quality education.

### Construction of early childhood and preschool education's comprehensive quality evaluation index system model

According to the practice of promoting quality-oriented education in schools in China, the experience of other kindergarten administrators, and the advice of pedagogy experts, the abovementioned design principles and methods were adopted in this study, including the case analysis, literature review, questionnaire survey, and mutual evaluation methods. A set of index systems suitable for the comprehensive quality evaluation of students in preschool education kindergarten was established (introduced in Table 1. Due to space limitations, the index system and data collection standards are combined into one table).

**Table 1:** Index system and collection standard.

**Level-1**	**Level-2**	**Level-3**
Ideological and moral quality	Political quality	Political attitude
		Political theory knowledge
		Party history
	Moral quality	Integrity
		Civilization etiquette
		Social responsibility
	Legal concept quality	Law-abiding
		Legal knowledge learning
Professional quality	Professional theory	Public basic course knowledge
		Specialized basic course knowledge
		Specialized special course knowledge
		Specialized laboratory courses
	Professional skills	Foreign language application ability
		Computer application ability
		Literature collection and retrieval ability
		Scientific and technological innovation practice (or artistic design practice)
	Penetration of arts and crafts education	Degree of professional integration
		Double degree
Physical and psychological quality	Psychological quality	Psychological health status
		Psychological course
	Physical quality	Physical health status
		Physical education grades
		Physical activities and sports competitions
Cultural quality	Cultural and artistic accomplishment	Club activities
		Various cultural competitions at all levels (competitions that can be
		participated by art and engineering students)
		Cultural and artistic cultivation knowledge assessment
	Artistic knowledge	Elective courses in arts
	Knowledge of humanities and social sciences	Elective courses in humanities and social sciences
	Knowledge of natural sciences	Elective courses in natural sciences
Ability quality	Organizational management capabilities	Interpersonal skills
		Management capabilities
		Teamwork skills
	Academic research ability	Papers, patents, publications
	Scientific and technological innovation capabilities	Various technology competitions
	Artistic innovation ability	Art competitions

In the propsed index system,the comprehensive evaluation index system in this paper includes both qualitative and quantitative indicators. In the index system of this paper, quantifiable quantitative indicators are given priority. Secondly, this paper also combines some necessary qualitative indicators as the composition of the comprehensive index system. Table 1 specifically introduces the index system we put forward.

### Weightage table of the indicator system

In this study, the analytic hierarchy process (AHP) was used to further calculate the whole index system to obtain the weightage of the index system proposed here. The calculation process and the consistency test process of AHP are not described here. Table 2 shows the weightage table of the indicator system.

**Table 2:** Weightage table of the indicator system.

**Level-1**	**Level-2**
Ideological and moral quality *U*_1_ 0.1599	Political quality *U*_11_ 0.07
	Moral quality *U*_12_ 0.086
	Legal concept quality *U*_13_ 0.044
Professional quality *U*_2_ 0.3185	Professional theory *U*_21_ 0.1248
	Professional skills *U*_22_ 0.128
	Penetration of arts and crafts education *U*_23_ 0.0672
Physical and psychological quality *U*_3_ 0.0973	Psychological quality *U*_31_ 0.072
	Physical quality *U*_32_ 0.048
Cultural quality *U*_4_ 0.1618	Cultural and artistic accomplishment *U*_41_ 0.048
	Artistic knowledge *U*_42_ 0.03
	Humanities and social sciences knowledge *U*_43_ 0.0405
	Natural sciences knowledge *U*_44_ 0.0315
Ability quality *U*_5_ 0.2625	Organizational management capabilities *U*_51_ 0.0714
	Academic research ability *U*_52_ 0.0567
	Scientific and technological innovation capabilities *U*_53_ 0.0875
	Artistic innovation ability *U*_54_ 0.0875

## Fuzzy comprehensive evaluation method of early childhood

### Single-level fuzzy comprehensive evaluation

1. Determine the set of evaluation indicators *U* = {*u*_1_, *u*_2_, ⋯ , *u*_*m*_}, where *u*_*i*_(*i* = 1, 2, ⋯ , *m*) is the evaluation index of the same level.
2. Determine the membership matrix.
3. Suppose that a one-factor evaluation of the *i*th evaluation indicator *u*_*i*_ yields a fuzzy vector *R*_*i*_ = (*r*_*i*1_, *r*_*i*2_, ⋯ , *r*_*im*_) relative to the comment set *V*, and *r*_*ij*_ is the degree to which the indicator *u*_*i*_ has *v*_*j*_, 0 ≤ *r*_*ij*_ ≤ 1. If the *m* indicator is comprehensively evaluated, the result is a matrix $R=\left[\begin{array}{c} \begin{array}{cc} r_{11} & r_{12}\\ r_{21} & r_{22} \end{array}\;\begin{array}{cc} \cdots & r_{1n}\\ \cdots & r_{2n} \end{array}\\ \begin{array}{cc} \cdots & \cdots \\ r_{m1} & r_{m2} \end{array}\;\begin{array}{cc} \cdots & \cdots \\ \cdots & r_{mn} \end{array} \end{array}\right]$ of *m* rows *n* columns (fuzzy relationship matrix).
4. Determine the weight vector *W* = (*w*_1_, *w*_2_, ⋯ , *w*_*m*_), where *w*_*i*_(*i* = 1, 2, ⋯ , *m*) indicates the importance of the indicator *u*_*i*_.
5. The fuzzy comprehensive evaluation results are obtained.

The fuzzy synthesis of the weight vector and the membership matrix yields a fuzzy evaluation result set, i.e.,


(1) $$\begin{array}{r} B=W\circ R=W\circ {\left(R_{1},R_{2},\cdots \;,R_{m}\right)}^{T}\\ =\left\{w_{1},w_{2},{\cdots \;,w}_{m}\right\}\circ \left[\begin{array}{c} \begin{array}{cc} r_{11} & r_{12}\\ r_{21} & r_{22} \end{array}\;\begin{array}{cc} \cdots & r_{1n}\\ \cdots & r_{2n} \end{array}\\ \begin{array}{cc} \cdots & \cdots \\ r_{m1} & r_{m2} \end{array}\;\begin{array}{cc} \cdots & \cdots \\ \cdots & r_{mn} \end{array} \end{array}\right]\\ =\left(b_{1},b_{2},{\cdots \;,b}_{n}\right), \end{array}$$

where, $b_{j}=\overset{m}{\underset{i=1}{\sum }}w_{i}\;\circ r_{ij},\;j=\;1,2,\cdots \;,n$.

### Multi-level fuzzy comprehensive evaluation

A multi-level fuzzy comprehensive evaluation should be used when the following situations occur.

1. There are many elements in the evaluation indicator set *U*, and the weight coefficient is difficult to determine. At this time, all indicators are generally divided into several categories according to their nature, and each category is first evaluated in a fuzzy and comprehensive manner, and then, a general fuzzy comprehensive evaluation is carried out. If each type of indicator can be reclassified, such a judgment can be carried out many times.
2. There are multiple levels of indicators in the evaluation indicator set *U*, that is, an indicator is often determined by several other indicators. At this time, the low-level indicators are commonly comprehensively evaluated first, and then, the indicators at the previous level are comprehensively evaluated.
3. The indicators in the evaluation indicator set *U* are ambiguous. At this time, the indicators in U are generally divided into several levels according to their nature, and the evaluation of the indicators is realized through the comprehensive evaluation of several levels of an indicator, and then a comprehensive evaluation of all indicators is carried out.

Steps of a multi-level fuzzy comprehensive evaluation algorithm:

1) Determine the hierarchical relationship between indicators.

The first level indicator set is *U* = {*u*_1_, *u*_2_, ⋯ , *u*_*m*_}, and the second level indicator set contained in the first level indicator *u*_*i*_ is *U*_*i*_ = {*u*_*i*1_, *u*_*i*2_, ⋯ , *u*_*ip*_}, *i* = 1, 2, ⋯ , *m*.

2) Construct a weight set of indicators at all levels.

According to the importance of indicators at each level, each level of indicators is given a corresponding weight coefficient. For example, the first level of the indicator weight coefficient set is *W* = (*w*_1_, *w*_2_, ⋯ , *w*_*m*_), where *w*_*i*_(*i* = 1, 2, ⋯ , *m*) indicates the importance of the indicator *u*_*i*_.

The set of the weight coefficients of the second level indicator contained in *u*_*i*_ in the first layer indicator is *W*_*ij*_ = (*w*_*i*1_, *w*_*i*2_, ⋯ , *w*_*ip*_), where *w*_*ij*_(*i* = 1, 2, ⋯ , *m, j* = 1, 2, ⋯ , *p*) indicates the importance of the indicator *u*_*ij*_.

3) Construct a comment set *V* = {*v*_1_, *v*_2_, ⋯ , *v*_*n*_}, where *v*_*j*_(*j* = 1, 2, ⋯ , *n*) is the evaluation level, which is the same as the meaning of the comment set in the single-level fuzzy comprehensive evaluation.
4) Fuzzy comprehensive evaluation of the second level.

A comprehensive evaluation is carried out by indicators in a certain category of the second level. If the *j*(*j* = 1, 2, ⋯ , *p*) indicator in the *i*(*i* = 1, 2, ⋯ , *m*) class is a fuzzy comprehensive evaluation, and the evaluation object belongs to the *k*(*k* = 1, 2, ⋯ , *n*) indicator in the comment set with the membership degree of *r*_*ijk*_(*i* = 1, 2, ⋯ , *m, j* = 1, 2, ⋯ , *p, k* = 1, 2, ⋯ , *n*), then the membership matrix of the *i*(*i* = 1, 2, ⋯ , *m*) class indicators are:


(2) $$\begin{array}{r} R_{i}=\left[\begin{array}{c} \begin{array}{cc} r_{i11} & r_{i12}\\ r_{i21} & r_{i22} \end{array}\;\begin{array}{cc} \cdots & r_{i1n}\\ \cdots & r_{i2n} \end{array}\\ \begin{array}{cc} \cdots & \cdots \\ r_{ip1} & r_{ip2} \end{array}\;\begin{array}{cc} \cdots & \cdots \\ \cdots & r_{ipn} \end{array} \end{array}\right] \end{array}$$

Then, the fuzzy synthesis evaluation set of *i*(*i* = 1, 2, ⋯ , *m*) indicator is:


(3) $$\begin{array}{r} B_{i}=W_{i}\circ R_{i}=W_{i}\circ {\left(R_{i1},R_{i2},\cdots \;,R_{im}\right)}^{T}\\ =\left\{w_{i1},w_{i2},{\cdots \;,w}_{im}\right\}\circ \left[\begin{array}{c} \begin{array}{cc} r_{i11} & r_{i12}\\ r_{i21} & r_{i22} \end{array}\;\begin{array}{cc} \cdots & r_{i1n}\\ \cdots & r_{i2n} \end{array}\\ \begin{array}{cc} \cdots & \cdots \\ r_{ip1} & r_{ip2} \end{array}\;\begin{array}{cc} \cdots & \cdots \\ \cdots & r_{ipn} \end{array} \end{array}\right]\\ =\left(b_{i1},b_{i2},{\cdots \;,b}_{in}\right) \end{array}$$

5) Fuzzy comprehensive evaluation of the first level.

The second level of a fuzzy comprehensive evaluation is only a vague synthesis of the types of the second level; to obtain the first level of a comprehensive evaluation, it is necessary to carry out a fuzzy synthesis between the types of the second level. The fuzzy evaluation vectors obtained from the evaluation of the second level of various types are regarded as the one-factor fuzzy evaluation vectors corresponding to the first level of indicators so that the fuzzy comprehensive evaluation of the first level can still be regarded as a single-level fuzzy comprehensive evaluation, and the membership matrix of the first level indicators is composed of the fuzzy comprehensive evaluation vectors of the second level, that is:


(4) $$\begin{array}{r} R=\left(\begin{array}{c} B_{1}\\ B_{2}\\ \cdots \\ B_{m} \end{array}\right)=\left[\begin{array}{c} \begin{array}{cc} b_{11} & b_{12}\\ b_{21} & b_{22} \end{array}\;\begin{array}{cc} \cdots & b_{1n}\\ \cdots & b_{2n} \end{array}\\ \begin{array}{cc} \cdots & \cdots \\ b_{m1} & b_{m2} \end{array}\;\begin{array}{cc} \cdots & \cdots \\ \cdots & b_{mn} \end{array} \end{array}\right] \end{array}$$

The set of the first level fuzzy comprehensive evaluation results is as follows:


(5) $$\begin{array}{r} B=W\circ R=\left\{w_{1},w_{2},{\cdots \;,w}_{m}\right\}\circ \left[\begin{array}{c} \begin{array}{cc} b_{11} & b_{12}\\ b_{21} & b_{22} \end{array}\;\begin{array}{cc} \cdots & b_{1n}\\ \cdots & b_{2n} \end{array}\\ \begin{array}{cc} \cdots & \cdots \\ b_{m1} & b_{m2} \end{array}\;\begin{array}{cc} \cdots & \cdots \\ \cdots & b_{mn} \end{array} \end{array}\right]\\ =\left(b_{1},b_{2},{\cdots \;,b}_{n}\right), \end{array}$$

where, $b_{j}=\overset{m}{\underset{i=1}{\sum }}w_{i}\;\circ b_{ij},\;j=\;1,2,\cdots \;,n$.

For fuzzy comprehensive evaluation methods above two levels, the basic idea is similar to the fuzzy comprehensive evaluation methods at two levels.

### Fuzzy evaluation model of comprehensive quality of early childhood and preschool education based on analytic hierarchy

On the basis of determining the comprehensive quality evaluation index system of early childhood and preschool education and the weights of each index in the previous article, a multi-level fuzzy comprehensive evaluation of the evaluation index system was carried out. Table 1 divides the factors affecting the comprehensive quality of early childhood and preschool education into three layers.

1. Determine the comment level *V* = {*v*_1_, *v*_2_, ⋯ , *v*_*n*_}, where *v*_*j*_(*j* = 1, 2, ⋯ , *n*) is the evaluation level, indicating the comments at all levels from high to low. In this s, we took *n* = 5 to evaluate the evaluation indicators of the comprehensive quality of early childhood and preschool education, and the comment set of the grade set established by the evaluation is *V* = {*v*_1_, *v*_2_, *v*_3_, *v*_4_, *v*_5_} = {*excellent, good, general, passing, poor* }.
2. Establish a fuzzy relationship matrix. The construction of the fuzzy relationship matrix is one of the most critical links in the comprehensive evaluation model, and whether its setting is in line with the actual situation has a great impact on the evaluation results. The general principle it established was to have conformity with reality as the standard, proceed from the specific characteristics of the ambiguity phenomenon, and pay attention to summarizing and absorbing the practical experience accumulated by people for a long time, especially the experience of experts. In the comprehensive quality evaluation index system of early childhood and preschool education, qualitative and quantitative indicators were included. Moral quality, psychological quality, cultural quality, etc., were included as qualitative indicators. Professional quality and similarly other related parameters were included as quantitative indicators. Different types of indicators have different methods of constructing their membership vectors.
  In the specific research of this study, the type of indicator was defined as a quantitative indicator, that is, the specific evaluation of a certain indicator was carried out by using the method of scoring on a percentage system (the score value was between 0 and 100 points).
3. Determination of membership functions and judgment matrices.

In the systematic evaluation of the comprehensive quality of early childhood and preschool education studied in this study, because the fuzzy matrix operation was obtained as a fuzzy vector, it cannot be used directly for the ranking evaluation of the results, so the author assigned a vector *S* to each comment level to indicate the set of comment levels, such as “excellent” is 90, “good” is 80, “general” is 70, “passing” is 60, and “poor” is 50, then *S* = [90, 80, 70, 60, 50]. According to the characteristics of the comprehensive quality evaluation of early childhood and preschool education, this study used the widely used triangle membership function to determine the membership of each grade to eliminate the unreasonable phenomenon caused by the transition of adjacent grades. The data of the midpoint was blurred, that is, the midpoint of each grade interval was used as the demarcation point. When the indicator entered the midpoint of the interval, the indicator belonged to the grade as 1, and when the indicator entered the midpoint of the adjacent interval, the membership of the grade was 0, that is, *u*_*vi*_(*u*_*i*_) = 1, *u*_*vi*−1_(*u*_*i*−1_) = 0. According to the characteristics of the indicator, the formulation of its membership functioned as a linear function, linking the membership function of a certain indicator of 1 and 0 in the interval (or the corresponding comment *V* of the interval). The membership function of each level can be determined thus. The operation process used the following formula:


(6) $$\begin{array}{l} u_{v1}\left(u_{i}\right)=\{\begin{array}{c} 1,u_{i}s\geq 95\%\\ 10^{*}\left(85\%-u_{i}\right),85\%\leq u_{i}<95\%\\ 0,\;u_{i}<85\%\; \end{array}\\ u_{v2}\left(u_{i}\right)=\{\begin{array}{c} 10^{*}\left(u_{i}-75\%\right),75\%\leq u_{i}<85\%\\ 10^{*}\left(95\%-u_{i}\right),85\%\leq u_{i}<95\%\\ 0,u_{i}<75\%\;or\;u_{i}\geq 95\% \end{array}\\ u_{v3}\left(u_{i}\right)=\{\begin{array}{c} 10^{*}\left(u_{i}-65\%\right),65\%\leq u_{i}<75\%\\ 10^{*}\left(85\%-u_{i}\right),75\%\leq u_{i}<85\%\\ 0,u_{i}<65\%\;or\;u_{i}\geq 85\% \end{array}\\ u_{v4}\left(u_{i}\right)=\{\begin{array}{c} 10^{*}\left(u_{i}-55\%\right),55\%\leq u_{i}<65\%\\ 10^{*}\left(75\%-u_{i}\right),65\%\leq u_{i}<75\%\\ 0,u_{i}<55\%\;or\;u_{i}\geq 75\% \end{array}\\ u_{v5}\left(u_{i}\right)=\{\begin{array}{c} 0,u_{i}\geq 65\%\\ 10^{*}\left(75\%-u_{i}\right),65\%\leq u_{i}<75\%\\ 1,\;u_{i}<55\%\; \end{array} \end{array}$$

In the end, all the factors of *U* are combined into a matrix:


(7) $$\begin{array}{r} B=A^{*}R=A^{*}{\left(R_{1},R_{2},\cdots \;,R_{m}\right)}^{T}\\ =\left\{a_{1},a_{2},{\cdots \;,a}_{m}\right\}\left[\begin{array}{c} \begin{array}{cc} r_{11} & r_{12}\\ r_{21} & r_{22} \end{array}\;\begin{array}{cc} \cdots & r_{1n}\\ \cdots & r_{2n} \end{array}\\ \begin{array}{cc} \cdots & \cdots \\ r_{m1} & r_{m2} \end{array}\;\begin{array}{cc} \cdots & \cdots \\ \cdots & r_{mn} \end{array} \end{array}\right] \end{array}$$

*R*_*i*_ is the fuzzy mapping of one-factor decision-making, *B* is the comprehensive evaluation matrix of all factors, *m* is the number of elements of the factor set, *n* is the number of elements of the evaluation set, and *i* is the factor marker that plays a role in the factor set, and then the judgment matrix *R* is obtained.

## Comprehensive quality evaluation model for early childhood and preschool education based on FNN

### Combination model of artificial neural network and fuzzy system

In this study, the FNN system was constructed using fuzzy reasoning.

1. According to the study of comprehensive quality knowledge, the fuzzy subset and fuzzy rules of the fuzzy system are preliminarily determined so as to construct a preliminary fuzzy system model.
2. According to the preliminarily determined fuzzy system model, the membership function and rules of a fuzzy system are constructed and its connection mode and weights are determined.
3. The identified fuzzy system model was applied to the actual FNN model.
4. The data obtained from each indicator was used to train and learn the preliminarily determined fuzzy neural network system to improve its accuracy.
5. The connection weight in the learned FNN system can be changed by changing the membership function and the fuzzy rule. The fuzzy system model can be further modified accordingly so as to construct a more accurate fuzzy neural network model.

### Research on the comprehensive quality model of early childhood and preschool education based on FNN

The comprehensive quality evaluation of early childhood and preschool education is a typical pattern recognition outcome, and the fuzzy neural network has a strong superiority in dealing with such outcomes. Therefore, it is a novel idea and a new method to apply fuzzy neural networks in the evaluation of early childhood and preschool education's comprehensive quality.

#### Determination of the FNN comprehensive quality evaluation method

To establish the FNN method of comprehensive quality evaluation of early childhood and preschool education, in this study, an FNN consisting of one input layer, one output layer, and two hidden layers is proposed.

#### Selection of the membership function in the FNN model

The second layer of the FNN comprehensive quality evaluation model is the membership function generation layer.

The commonly used membership functions are listed as follows:

(1) Triangular function.
  Its characteristic is simple structure, but the classification was relatively rough.
(2) The Gaussian function.

$\mu_{ij}=exp(-{\left(\frac{x_{i}-c_{ij}}{\sigma_{ij}}\right)}^{2}$, where *c*_*ij*_ represents the center of the membership function and σ_*ij*_ determines the radius of the membership function.

Its characteristics are good local characteristics, fast convergence speed, and easy ecommunication of fuzzy knowledge, but the structure is more complex.

The comprehensive quality evaluation of early childhood and preschool education has high requirements for classification, and the triangle membership function has high errors in expressing fuzzy knowledge. Based on the above analysis and comparison, the Gaussian-type membership function was employed for FNN in this article.

#### Determination of the fuzzy rules in the FNN model

To diminish the reliance of the learning results on the spatial dispersion of students' quality examples and further develop the assembly rate, the K-means strategy was taken on to ascertain the pertinence of the standards directly from the info tests. According to the results of early childhood and preschool education's various grades, the sample set was divided into five categories, namely: excellent, good, general, passing, and poor. The idea is to renew the value of each cluster center successively by an iterative method until the best clustering results are obtained. The specific algorithm is as follows:

1. Seek the average matrix M of each student's comprehensive quality indicator.
2. Aggregate the matrix M 37 times vertically (37 comprehensive quality indicators) and one time horizontally.
3. In the 37th aggregation, seek any sample from the distance to 5 centers and classify the sample to the class of the center with the shortest distance;
4. Update the central value of the class with the mean method;
5. For all 5 cluster centers, if the value remains unchanged with the iteration method of (3) (4), the iteration will end; otherwise, the iteration will continue.

### Learning algorithm analysis of FNN comprehensive quality evaluation

In our study, the fuzzy segmentation number of early childhood and preschool education had been set by the K-means. They were excellent, good, general, passing, and poor. Nevertheless, the parameters to be learned were mainly the connection weight *w*_*ij*_(*i* = 1, 2, ⋯ , *r*; *j* = 1, 2, ⋯ , *h*) of the last layer, the central value *c*_*ij*_ of the membership function of the second layer, and the width σ_*ij*_(*i* = 1, 2, ⋯ , *n*; *j* = 1, 2, ⋯ , *h*) of the membership function. FNN is also a multi-layer feedforward network. In this study, the BP network learning model was used to adjust parameters by error backpropagation. To derive the iterative algorithm of error backpropagation, the input and output relationships of each neuron were formally described.

#### Basic structure of individual neurons

**Figure 1:**
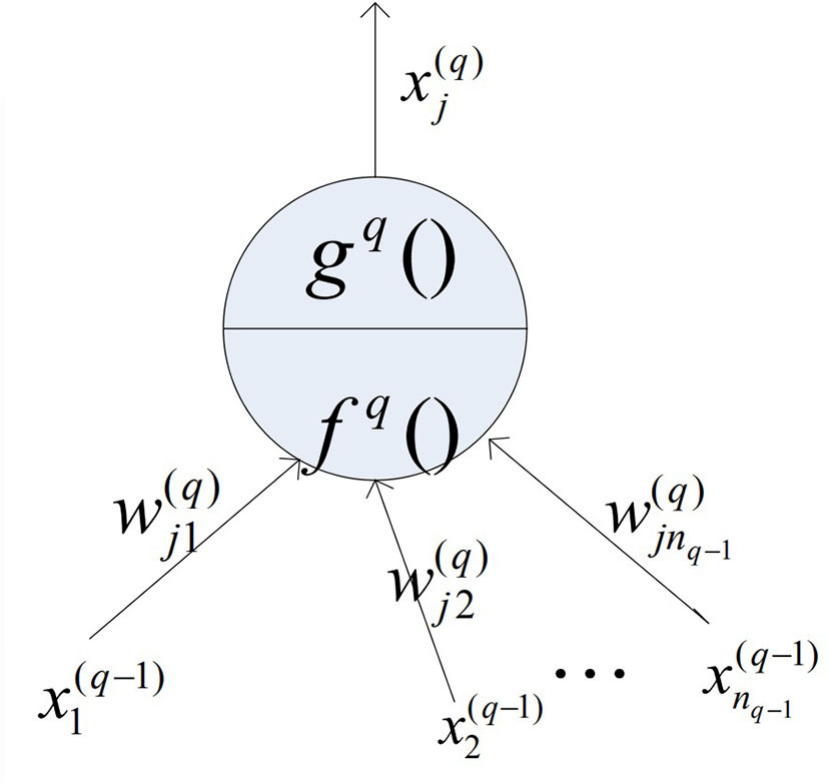
Neuronal node structure.

In order to analyze the relationship of input and output in each layer of the FNN, the structure diagram of individual neurons was introduced. Figure 1 shows the input and output relationships of layer *q* and node *j* in the fuzzy neural network,

where the pure input of the node is $f^{\left(q\right)}\left(x_{1}^{\left(q-1\right)},x_{2}^{\left(q-1\right)},\cdots \;,x_{n_{q-1}}^{\left(q-1\right)};w_{j1}^{\left(q\right)};w_{j2}^{\left(q\right)},\cdots \;,w_{jn_{q-1}}^{\left(q\right)}\right)$, and the output of the node is $x_{j}^{\left(q\right)}=\;g^{\left(q\right)}\;f^{\left(q\right)}$. For general neuron nodes, there are usually:


$$f^{\left(q\right)}=\sum_{i=1}^{n_{q-1}}w_{ji}^{\left(q\right)}x_{i}^{\left(q-1\right)}x_{j}^{\left(q\right)}=g^{\left(q\right)}\;(f^{\left(q\right)})=\frac{1}{1+e^{-\mu \;f^{\left(q\right)}}}$$

#### Input-output relationship of each layer of comprehensive quality evaluation based on FNN

For the FNN model of college student comprehensive quality evaluation, the input and output functions of neuron nodes have a special form. The node functions of each layer are given in detail below.

The first layer is the input layer. Input comprehensive quality evaluation indicators are given in (37).


(8) $$\begin{array}{r} \text{Input:}f_{i}=x_{i}^{\left(0\right)}=x_{i} \end{array}$$

(9) $$\begin{array}{r} \text{Output:}x_{i}^{\left(1\right)}=g_{i}^{\left(1\right)}=f_{i}^{\left(1\right)};i=1,2,\cdots \;,n \end{array}$$

The second layer is the membership function generation layer.


(10) $$\begin{array}{r} \text{Input:}f_{ij}^{\left(2\right)}=-\frac{{\left(X_{i}^{\left(1\right)}-C_{ij}\right)}^{2}}{\sigma_{ij}^{2}} \end{array}$$

(11) $$\begin{array}{r} \text{Output:}x_{ij}^{\left(2\right)}=\mu_{ij}=g_{ij}^{\left(2\right)}=exp\left(-\frac{{\left(X_{i}^{\left(1\right)}-C_{ij}\right)}^{2}}{\sigma_{ij}^{2}}\right) \end{array}$$

The third layer is the reasoning layer.


(12) $$\begin{array}{r} \text{Input:}f_{j}^{\left(3\right)}=x_{{1j}_{1}}^{\left(2\right)}x_{{2j}_{2}}^{\left(2\right)}\cdots x_{nj_{n}}^{\left(2\right)} \end{array}$$

(13) $$\begin{array}{r} \text{Output:}x_{j}^{\left(3\right)}=g_{j}^{\left(3\right)}=f_{i}^{\left(4\right)} \end{array}$$

The fourth layer is the anti-fuzzification layer.


(14) $$\begin{array}{r} \text{Input: }f_{i}^{\left(4\right)}=\overset{m}{\underset{j=1}{\sum }}w_{ij}x_{j}^{\left(3\right)} \end{array}$$

(15) $$\begin{array}{r} \text{Output: }x_{i}^{\left(4\right)}=y=g_{i}^{\left(4\right)}=f_{i}^{\left(4\right)}\;i=1,2,\cdots \;,r \end{array}$$

The above layers were the forward calculation process of the FNN, calculating the input and output values of corresponding nodes in each layer of the model.

#### Parameter adjustment of FNN model

Let the error energy function of the network be


(16) $$\begin{array}{r} E=\frac{1}{2}\overset{r}{\underset{i=1}{\sum }}{\left(y_{di}-y_{i}\right)}^{2}, \end{array}$$

where *y*_*di*_ is the actual output value of comprehensive quality, *y*_*i*_ is the desired output value, and *E* is the squared error function. The error back propagation algorithm is used to push down $\frac{\partial E}{\partial w_{ij}}$, $\frac{\partial E}{\partial c_{ij}}$, and $\frac{\partial E}{\partial \sigma_{ij}}$ , and then, the one-step optimization method is used to adjust *w*_*ij*_, *c*_*ij*_, and σ_*ij*_.

(1) Calculate the back propagation error of the fourth anti-fuzzy layer.
(17) $$\begin{array}{r} \delta_{i}^{\left(4\right)}=-\frac{\partial E}{\partial f_{i}^{\left(4\right)}}=-\frac{\partial E}{\partial y_{i}}=y_{di}-y_{i} \end{array}$$
  Then obtain:
(18) $$\begin{array}{r} \frac{\partial E}{\partial w_{ij}}=\frac{\partial E}{\partial f_{i}^{\left(4\right)}}\frac{\partial f_{i}^{\left(4\right)}}{\partial w_{ij}}=-\delta_{i}^{\left(4\right)}x_{j}^{\left(3\right)} \end{array}$$
(2) Calculate the back propagation error of the third inference layer.
(19) $$\begin{array}{r} \delta_{j}^{\left(3\right)}=-\frac{\partial E}{\partial f_{j}^{\left(3\right)}}=\frac{\partial E}{\partial f_{i}^{\left(4\right)}}\frac{\partial f_{i}^{\left(4\right)}}{{\partial g}_{j}^{\left(3\right)}}\frac{{\partial g}_{j}^{\left(3\right)}}{{\partial f}_{j}^{\left(3\right)}}\\ =\delta_{j}^{\left(4\right)}\overset{m}{\underset{i=1,i\neq j}{\sum }}x_{i}^{\left(3\right)}/{\left(\overset{m}{\underset{i=1}{\sum }}x_{i}^{\left(3\right)}\right)}^{2} \end{array}$$
Calculate the back propagation error of the second membership function layer.

(20) $$\begin{array}{r} \delta_{j}^{\left(2\right)}=-\frac{\partial E}{\partial f_{ij}^{\left(2\right)}}=-\overset{m}{\underset{k=1}{\sum }}\frac{\partial E}{\partial f_{k}^{\left(3\right)}}\frac{\partial f_{k}^{\left(3\right)}}{{\partial g}_{ij}^{\left(2\right)}}\frac{{\partial g}_{ij}^{\left(2\right)}}{{\partial f}_{ij}^{\left(2\right)}}\\ =\overset{m}{\underset{k=1}{\sum }}\delta_{k}^{\left(3\right)}S_{ij}exp\left(-\frac{{\left(x_{i}-C_{ij}\right)}^{2}}{\sigma_{ij}^{2}}\right) \end{array}$$

*f*^(3)^ in this study adopts multiplication operation, when $g_{ij}^{\left(2\right)}=\mu_{ij}$ is an input of the k-th regular node:


(21) $$\begin{array}{r} S_{ij}=\frac{\partial f_{k}^{\left(3\right)}}{{\partial g}_{ij}^{\left(2\right)}}=\frac{\partial f_{k}^{\left(3\right)}}{\partial \mu_{ij}}=\overset{n}{\underset{i=1,i\neq j}{\prod }}\mu_{ij} \end{array}$$

Otherwise, $S_{ij}=\frac{\partial f_{k}^{\left(3\right)}}{{\partial g}_{ij}^{\left(2\right)}}=\frac{\partial f_{k}^{\left(3\right)}}{\partial \mu_{ij}}=\;0$.

Thus, the adjustment amount from *c*_*ij*_, *b*_*ij*_, and *w*_*ij*_ in the learning process can be expressed by the following formula:


(22) $$\begin{array}{r} c_{ij}\left(n+1\right)-c_{ij}\left(n\right)=-\eta \frac{\partial E}{\partial c_{ij}}\\ =-\eta \frac{\partial E}{{\partial f}_{ij}^{\left(2\right)}}\frac{{\partial f}_{ij}^{\left(2\right)}}{c_{ij}}=-\delta_{ij}^{\left(2\right)}\frac{2\left(x_{i}-C_{ij}\right)}{\sigma_{ij}^{2}}\\ i=1,2,\cdots \;,nj=1,2,\cdots \;,m_{i} \end{array}$$

(23) $$\begin{array}{r} \sigma_{ij}\left(n+1\right)-\sigma_{ij}\left(n\right)=-\eta \frac{\partial E}{\partial \sigma_{ij}}\\ =-\eta \frac{\partial E}{{\partial f}_{ij}^{\left(2\right)}}\frac{{\partial f}_{ij}^{\left(2\right)}}{\sigma_{ij}}=-\delta_{ij}^{\left(2\right)}\frac{2{\left(x_{i}-C_{ij}\right)}^{2}}{\sigma_{ij}^{2}}\\ i=1,2,\cdots \;,nj=1,2,\cdots \;,m_{i} \end{array}$$

According to the gradient descent method, the change term of weight (threshold) *w*_*ij*_ is proportional to ∂*E*/∂*w*_*ij*_.


(24) $$\begin{array}{l} w_{ij}\left(n+1\right)-w_{ij}\left(n\right)=w_{ij}=\;\eta \partial E/{\partial w}_{ij}\\ i=1,2,\cdots \;,r\;j=1,2,\cdots \;,m \end{array}$$

where η is the learning rate of the network, and its value ranges from 0 to 1.

## Prediction results of early childhood and preschool education's comprehensive quality based on FNN

### Improving the FNN algorithm of hidden layer nodes

The author proposes a method to improve the fuzzy neural network of hidden layer nodes. For the fuzzy neural network system determined in this study, the number of input and output sample space is P, that is, $x\left(m\right)={\left[x_{1}\left(m\right),x_{2}\left(m\right),\cdots \;,x_{n}\left(m\right)\right]}^{T}$ and *y*(*m*) , (*m* = 1, 2, ⋯ , *P*), and L is the number of nodes in the hidden layer. For the hidden layer, there is $z\left(m\right)={\left[z_{1}\left(m\right),z_{2}\left(m\right),\cdots \;,z_{n}\left(m\right)\right]}^{T},\;\left(m=1,2,\cdots \;,P\right)$, where $z_{l}=\mu_{{1j}_{1}}\mu_{{2j}_{2}}\cdots \mu_{nj_{n}}=\prod_{i=1}^{n}=exp\left[-{\left(\frac{x_{i}-c_{ij}}{\sigma_{ij}}\right)}^{2}\right]$ *l* = 1, 2, ⋯ , *L*

The similarity measure for input


(25) $$\begin{array}{r} x\left(m\right)isa_{ij}\left(N\right)=||x\left(i\right)-x\left(j\right)||; \end{array}$$

For the hidden layer *z*(*m*), the similarity measure is:


(26) $$\begin{array}{r} b_{ij}\left(N\right)=||z\left(i\right)-z\left(j\right)|| \end{array}$$

The similarity measure for the output *y*(*m*) is:


(27) $$\begin{array}{r} d_{ij}\left(N\right)=||y\left(i\right)-y\left(j\right)|| \end{array}$$

Nevertheless, the similarity measurement relation among input and hidden layer space is:


(28) $$\begin{array}{r} S_{1}\left(L\right)=\overset{N}{\underset{i=1}{\sum }}\overset{N}{\underset{j=1}{\sum }}\left(a_{ij}\ln\frac{a_{ij}}{b_{ij}}+b_{ij}\ln\frac{b_{ij}}{a_{ij}}\right) \end{array}$$

The similar calculation relation among the hidden layer space and the output space is:


(29) $$\begin{array}{r} S_{2}\left(L\right)=\overset{N}{\underset{i=1}{\sum }}\overset{N}{\underset{j=1}{\sum }}\left(b_{ij}\ln\frac{b_{ij}}{d_{ij}}+d_{ij}\ln\frac{d_{ij}}{b_{ij}}\right) \end{array}$$

The total similar calculation of fuzzy neural network is:


(30) $$\begin{array}{r} S\left(L\right)=S_{1}\left(L\right)+S_{2}\left(L\right) \end{array}$$

According to formula (30), the total similar calculation of FNN is:


(31) $$\begin{array}{l} S\left(L\right)=S_{1}\left(L\right)+S_{2}\left(L\right)=\sum_{i=1}^{N}\sum_{j=1}^{N}\left(a_{ij}\ln\frac{a_{ij}}{b_{ij}}+b_{ij}\ln\frac{b_{ij}}{a_{ij}}\right)\\ +\sum_{i=1}^{N}\sum_{j=1}^{N}\left(b_{ij}\ln\frac{b_{ij}}{d_{ij}}+d_{ij}\ln\frac{d_{ij}}{b_{ij}}\right)\\ =\sum_{i=1}^{N}\sum_{j=1}^{N}(a_{ij}\ln a_{ij}•a_{ij}\ln b_{ij}+2b_{ij}\ln b_{ij}•b_{ij}\ln a_{ij}\\ +b_{ij}\ln d_{ij}+d_{ij}\ln d_{ij}+d_{ij}\ln b_{ij}) \end{array}$$

In formula (31) given above, since *x*(*m*) and *y*(*m*) are only dependent on the input and output samples and have nothing to do with the number of *L*, it could be seen from the Equations (28), (29), and (30) that only *b*_*ij*_ is related to L, and because *b*_*ij*_ is Euclian-distance, with the gradual increase of *L*, *b*_*ij*_ also increases. Therefore, Equation (31) can be simplified as:


(32) $$\begin{array}{l} S\left(L\right)=\sum_{i=1}^{N}\sum_{j=1}^{N}(-a_{ij}\ln b_{ij}+2b_{ij}\ln b_{ij}-b_{ij}\ln a_{ij}-b_{ij}\ln d_{ij}\\ -d_{ij}\ln b_{ij})=\sum_{i=1}^{N}\sum_{j=1}^{N}[\ln b_{ij}\left(b_{ij}-a_{ij}-d_{ij}\right)\\ +b_{ij}\left(\ln b_{ij}-lna_{ij}-lnd_{ij}\right)] \end{array}$$

Due to *L∞b*_*ij*_, the relationship between *L* and *S*(*L*) could be supplanted by the relationship between *b*_*ij*_ and formula (32). According to the analytical formula (31), the relationship between *S*(*L*) and *L* satisfies that, when *L* increases gradually, *S*(*L*) decreases first and then increases. In this changing trend, one *L*_*p*_ could always be found to minimize the similarity measure *S*(*L*_*p*_ ).

### Simulation experiment

In this study, the parameters of early childhood and preschool education's comprehensive quality were trained by using the above rules, and the number of hidden layer nodes was changed by a certain step length. The estimated value with a small change of similarity measure was found first, and then the step length was reduced, and the maximum number of nodes was finally obtained. The results are introduced in Table 3.

**Table 3:** Calculation results of *S*(*L*) (unit: 10^3^).

**Number of hidden layer nodes**	**5**	**10**	**15**	**20**	**25**	**30**	**35**	**40**
Similarity measure	32.481	20.130	14.430	7.593	5.379	4.226	4.196	3.966
Number of hidden layer nodes	45	50	60	80	110	150	185	
Similarity measure	3.963	3.835	3.955	4.164	4.428	9.373	25.124	

*Based on the results of the above table, we chose 50 as the number of hidden layer nodes*.

### Result and analysis

In this study, we selected 10 preschool teachers as a sample. The predicted result pairs are shown in Table 4.

**Table 4:** The result of the experiment case.

**Teacher**	**Actual**	**The network**	**Relative**	**The network output**	**Relative**
**ID**	**evaluation**	**output result**	**Relative**	**result after changing**	
	**results**	**of FNN**		**the hidden layer node**	
1	93.54	94.375	0.835	94.052	0.412
2	82.39	80.562	1.828	82.456	0.66
3	77.85	76.621	1.229	75.854	1.996
4	65.78	65.061	0.719	66.08	0.2
5	88.19	87.473	0.617	88.654	0.464
6	67.52	68.124	0.604	67.683	0.163
7	72.85	74.032	1.182	72.505	0.345
8	58.96	58.076	0.884	58.802	0.158
9	52.42	50.864	1.556	50.332	2.088
10	63.88	65.693	1.831	65.047	1.167

It can be determined from the above table that the model proposed in this study can accurately evaluate the teaching quality of preschool education teachers, with a relatively small error. Further observation shows that, compared with the basic FNN, the improved method obtained a smaller relative error of 0.158, which indicated that our improved method was effective.

## Conclusion

Fuzzy neural networks can overcome the one-sidedness of neural networks to some extent. And the FNN operation is not completely a black-box operation. Experts or teaching administrators can adjust the fuzzy operation rules according to the real experience so that the blindness of NN can be solved to a certain extent. Therefore, it is considered that the application of FNN in the evaluation of early childhood and preschool education's comprehensive quality has made certain progress and is a beneficial attempt. Compared with other algorithms, this evaluation method has the characteristics of science, simplicity, and strong operability, and the model structure and method application prospect are broad. Finally, the number of hidden layer nodes was adjusted based on the principle of similarity measure and the effectiveness of this method was verified through an example. After analysis and comparison, the results show that the number of hidden layer nodes obtained by similarity measure can improve the speed of network convergence.

This study analyzed the problems and the factors that affect early childhood and preschool education's comprehensive quality, using the method of AHP, fuzzy evaluation, and FNN. It proposed the setting up of a more scientific method that also conforms to the current early childhood education in combination with the kindergarten evaluation index system, evaluation method, and model. More importantly, it solves the problem of the difficult evaluation of preschool education teachers' comprehensive quality and makes employers measure talents from the perspective of comprehensive quality.

## Data availability statement

The raw data supporting the conclusions of this article will be made available by the authors, without undue reservation.

## Author contributions

The author confirms being the sole contributor of this work and has approved it for publication.

## Conflict of interest

The author declares that the research was conducted in the absence of any commercial or financial relationships that could be construed as a potential conflict of interest.

## Publisher's note

All claims expressed in this article are solely those of the authors and do not necessarily represent those of their affiliated organizations, or those of the publisher, the editors and the reviewers. Any product that may be evaluated in this article, or claim that may be made by its manufacturer, is not guaranteed or endorsed by the publisher.
